# Un lymphome épidural primitif révélé par un syndrome de la queue de cheval

**DOI:** 10.11604/pamj.2013.16.64.3247

**Published:** 2013-10-23

**Authors:** Metoui Leila, Ajili Faida

**Affiliations:** 1 Hôpital militaire de Tunis, Service de Médecine Interne, Tunis, Tunisie

**Keywords:** Lymphome, syndrome de la queue de cheval, IRM, Lymphoma, Cauda equina syndrome, IRM

## Image en médecine

**Figure 1:**
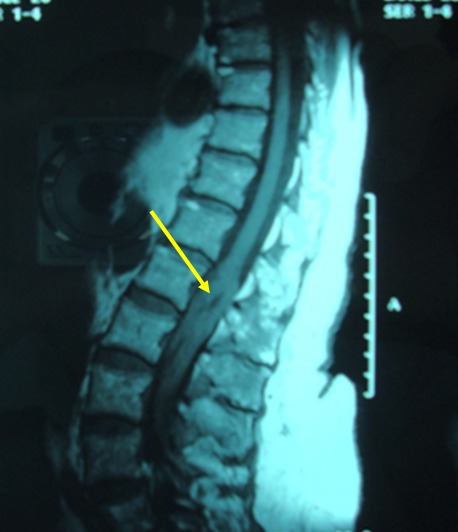
IRM en coupe sagittale montrant une lésion tissulaire extra-durale en regard de L2 L3 L4 en en hyper signal en T2 séquence T2

Le lymphome épidural primitif est une tumeur rare qui représente moins de 10% des tumeurs épidurales et moins de 1% des lymphomes non hodgkiniens. Le tableau clinique est souvent bruyant associant des rachialgies initiales puis des signes neurologiques déficitaires sensitifs (67% des cas) ou moteurs (90%), et exceptionnellement un syndrome de la queue de cheval. Nous rapportons l'observation d'une femme âgée de 82ans qui consulte devant une faiblesse des deux membres inférieurs et des troubles sphinctériens à type d'incontinence urinaire et de paresthésies de la zone périnéale (anovulaire). L'examen physique trouve une patiente en bon état général, une paraparésie avec hypoesthésie des deux membres inférieurs avec un niveau sensitif L4, une anesthésie en selle et des troubles de la sensibilité profonde. Il n'y a pas de syndrome tumoral. L'IRM médullaire a révélé une lésion tissulaire extra-durale en regard de L2 L3 L4 en iso signal en T1 et en hyper signal en T2. Une laminectomie au niveau de L2 L3 L4 réalisée a conclu à un lymphome non-hodgkinien à grandes cellules de phénotype B. Un bilan d'extension de la maladie pratiquée a montré une atteinte méningée. Le diagnostic de lymphome épidural primitif avec localisation méningée a été retenu. La patiente a reçu 4 cures de Chimiothérapie (MINI CEOP) selon le protocole LNH08 groupe 5 avec une excellente réponse clinique et radiologique. Le pronostic du lymphome épidural primitif est relativement bon à cinq ans avec une survie globale de 70% une survie sans maladie de 50%. Figure 1.

